# Phosphates in medications: Impact on dialysis patients 

**DOI:** 10.5414/CN109853

**Published:** 2020-02-12

**Authors:** Dixie-Ann Sawin, Lin Ma, Amanda Stennett, Norma Ofsthun, Rainer Himmele, Robert J. Kossmann, Franklin W. Maddux

**Affiliations:** 1 Fresenius Medical Care North America, Renal Therapies Group, and; 2 Fresenius Kidney Care, Waltham, MA, USA

**Keywords:** phosphate, pill burden, dialysis, medications

## Abstract

Maintaining phosphorus balance in in-center hemodialysis (ICHD) patients is problematic despite recommended dietary restriction, dialysis, and phosphate binder use. Rarely is P content in prescribed medications considered, but this source should raise concern. Data was obtained from the Fresenius Kidney Care (FKC) electronic data warehouse Knowledge Center and MedReview-eRx accessed Surescripts, housing > 80% of US-filled prescriptions. Adult FKC ICHD patients prescribed ≥ 1 medication in the MedReview-eRx database were analyzed (695,759 prescriptions). Information collected included medication dose, dose unit, dose timing, strength, start and stop dates, refills, demographic information, admission history, and modality type. Numbers of patients, prescriptions by individual medication, and drug class were then analyzed. Medications prescribed > 100 times were reported. Median doses/day (number of tablets) were calculated for each medication (open order on randomly selected day). Phosphate content of medications taken in FKC clinics was assessed using routinely used pharmacology references, and potential resulting phosphate and pill burden were also calculated. The top five prescribed drug classes in FKC dialysis patients were calcium-channel blockers (22%), proton pump inhibitors (PPIs; 18%), acetaminophen-opioid (AO; 13%), angiotensin-converting enzyme inhibitors (ACEi; 10%), and α2-agonists (9%). The maximum phosphate added for different medications varied by manufacturer. For instance, at median daily doses, phosphate contributions from the top five medications prescribed were 112 mg for amlodipine, 116.2 mg from lisinopril, 6.7 mg from clonidine, 0 mg from acetaminophen, and 200 mg for omeprazole. Prescribing these together could increase the daily phosphate load by 428 mg, forcing the patient to exceed the recommended daily intake (RDI) with food and drink. Phosphate content in medications prescribed to HD patients can substantially contribute to the daily phosphate load and, in combination, may even exceed the daily recommended dietary phosphate intake. Healthcare providers should monitor all medications containing phosphate prescribed in order to minimize risk of uncontrolled hyperphosphatemia and poor adherence.

## Introduction 

Maintaining phosphorus balance in end-stage renal disease (ESRD) can be problematic despite the use of dialysis and phosphate binders [1]. According to the KDOQI Guidelines, dietary phosphate intake should be limited to 800 – 1,000 mg/day (adjusted for dietary protein needs) in ESRD patients with serum phosphate levels above 5.5 mg/dL [2]. When determining phosphate intake from dietary phosphate sources, phosphates present in medications are rarely, if at all, considered. Phosphates, especially dibasic calcium phosphate, are commonly incorporated as “inactive ingredients” into medications, serving as anti-adherents, binders, coatings, disintegrants, fillers, flavors, colors, lubricants, glidants, sorbents, preservatives, or sweeteners. However, it was suggested that they could contribute to a patient’s phosphate load or have iatrogenic effects. 

In two seminal papers, Sherman et al. [3, 4] examined the labels of 200 generic and branded medications commonly used in Dialysis Clinic Inc. (DCI) facilities across the USA. Of those, they reported that 11.5% contained added phosphate [23]. Since the actual phosphate content was not listed on any of the labels, they determined the respective amounts by spectroscopic analysis. They reported a wide range of phosphate content in medications, ranging from 1.4 mg/tablet in clonidine (Blue Point Laboratories, Dublin, Ireland) to 111.5 mg/tablet on 40 mg paroxetine (GSK, Philadelphia, PA, USA). Phosphate content also varied by manufacturer; for instance, paroxetine 20 mg from Apotex (Toronto, Canada) contained no phosphate, while the same product from Aurobindo (Hyderabad, India) contained 37.5 mg of phosphate. These trends have been observed by others [5, 6]. In addition, Sultana et al. [7] assessed phosphate content in 3,779 pharmaceutical products used in an Italian database of chronic kidney disease (CKD) patients. Of these, 266 compounds contained absorbable phosphate, and the phosphate was present as part of the active moiety (0.8%), as a counter-ion (8.3%), or in excipients (94.4%). Among those products with absorbable phosphate_,_ a range of phosphate intake from 4 to 41 mg/day per product was reported; patients in that study had up to 17 prescriptions. Nelson et al. [6] also did an analysis on 1,744 drug formulations (from 124 drugs) prescribed to hemodialysis (HD) patients in Canada. Like Sherman et al. [3, 4], they identified that 11% contained phosphates. Patients were prescribed between 10 to 18 pills/day with a median calculated phosphate load from medications of 111 mg/day. Notably, the type of phosphate present in these drug compounds is classified as inorganic; as such, all phosphate discussed in this paper is inorganic. Several studies suggest that inorganic phosphate is more readily absorbed compared to phosphate from organic sources [8, 9]. 

Regarding the bioavailability of phosphate from different calcium phosphate salts, Wendt and Rodenhutscord [10] reported that 100% of the phosphate from monosodium phosphates, 96% from anhydrous dibasic calcium phosphate, 91% from monodibasic calcium phosphate, and 86% from dihydrated dibasic calcium phosphate would be available for absorption. Calcium glycerophosphate, which could also be used as an excipient, was shown to be more soluble than calcium phosphates, with very high solubility at pH values between 3 and 7.5 [11]. 

The FDA lists 34 phosphate-containing chemicals that are used in approved drug products in the U.S. (www.accesssdata.fda.gov accessed December 29, 2014). Though the FDA guidance on over-the-counter labeling only requires that the names of inactive ingredients be listed and not the amounts, new findings suggest that phosphate contribution from medications, considered as “hidden”, should be cause for concern. 

## Materials and methods 

Data was obtained from the Fresenius Kidney Care (FKC) electronic data warehouse Knowledge Center including MedReview-eRx, which uses the same e-prescription program used in Acumen EHR, to access Surescripts, the central electronic clearing house for over 80% of prescriptions filled in the United States. Adult in-center hemodialysis (ICHD) patients from FKC dialysis clinics, who were prescribed at least one of these medications in existing MedReview-eRx database, were included in the analysis. The following information was collected: medication dose, dose unit, dose timing, strength, start and stop dates, refills, generic and brand name, patient’s demographic information, admission history, and modality type. The primary analyses included the frequency of medications prescribed, median doses per day, and known phosphate content in individual products. Median dose (number of tablets) per day was calculated for each medication that had an open order on a randomly selected day, and only the medications with > 100 orders across the FKC ICHD population in 1 year were included in this investigation. In order to identify the phosphate content of medications prescribed in FKC clinics, we searched label information, as well as pharmacology references on compounding and manufacturing of medications. Most tablets prescribed in the U.S. are manufactured by compression methods, involving use of high pressures, steel punches, stamps, and dies to form powders or granulations. The formulations identified, including phosphate content, were obtained from compression methods used in drug manufacturing compliant with current good manufacturing practices (cGMP) and that allow consistent production of a given product (i.e., process validation and verification). To obtain accurate phosphate content values, information obtained from published experiments, data and analytical results that support the master formula, the in-process and ﬁnished product speciﬁcations, and the ﬁled manufacturing process were compiled. 

We also calculated the additional phosphate binder pill burden due to individual medications as follows: 


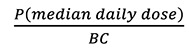
 Equation 1 

Where P is the phosphate contribution from the individual medication based on median daily dose, and BC is the phosphate binder binding capacity. 

## Results 

There were 209,811 unique in-center hemodialysis patients who were prescribed at least 1 medication in our study. The characteristics of this population are shown in Table 1. 

**Figure 1.**
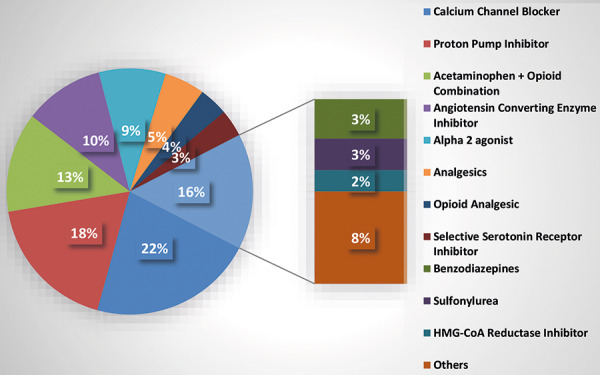
Percent of medications by drug class prescribed in Fresenius Kidney Care dialysis clinics (N = 695,759 orders).

From this population, we identified that the most prescribed drug classes from 695,759 physician orders in FKC in-center dialysis clinics was calcium channel blockers (21.66%), followed by proton pump inhibitors (PPIs, 18.11%), acetaminophen (13.1%), angiotensin converting enzyme (ACE) inhibitors (10.5%), and α2-agonists (8.8%) (Figure 1). 

Table 2 lists the prescribed medications and their respective contributions to the potential phosphate burden in mg/day for each medication based on the median dose (number of tablets) prescribed per day and phosphate bioavailability (i.e., 86 – 96% of phosphate present). The most frequently prescribed medication, amlodipine (12,742 orders), with a median dose of 1 tablet/day, would potentially increase the patient’s phosphate burden by 3.6 – 112 mg/day in patients taking this drug. Again, depending on the manufacturer, lisinopril – the second most prescribed medication – can contain anywhere from 3.6 to 121 mg/tablet. Thus, the amount of bioavailable phosphate potentially absorbed by the patient per day would range from 3.1 to 116.2 mg. Clonidine, which is the third most prescribed medication, could increase the phosphate burden by ~2.4 – 6.72 mg/day. Since acetaminophen does not contain any form of phosphate, it would not be expected to increase the daily phosphate burden, unless it was compounded with codeine. The fifth most prescribed medication, omeprazole, however, has a large amount of mono sodium phosphate added during the manufacturing process, 175 – 200 mg per tablet. At a median dose of 1 tablet/day, taking omeprazole could increase the phosphate burden by up to 200 mg since its phosphate is 100% bioavailable. Regarding other PPIs, esomeprazole and pantoprazole had no additional phosphate according to their prescribing information. However, compounding information suggests addition of calcium glycerophosphate to pantoprazole during manufacturing processes [12]. Although diphenhydramine was only prescribed 469 times in the period assessed, it is notable that the additional phosphate could be as high as 270 mg per day. Other major contributors to the daily phosphate burden identified include diclofenac, paroxetine, sildenafil, phenytoin, and alprazolam. Of these, paroxetine scripts made up 3.6% of all orders, alprazolam accounted for 3.2%, and sildenafil, phenytoin, and diclofenac were each prescribed in less than 0.5% of instances. Table 2 also shows the range of phosphate present in and the respective potential phosphate burden contributed by the most frequently prescribed medications according to ranges used in manufacturing. 

We also collated the data on the median phosphate binder frequency and dose (Table 3). Complete binder dosing information was available for 114,318 patients (54%). The remaining 46% of patients had no binder documented or data entry errors such as incorrect entry of medication name or missing dose and/or frequency. Since many of those patients may have been receiving phosphate binders, the following counts underestimate true percentages. 

Sevelamer (carbonate or hydrochloride) was prescribed in over 65,000 patients (29%), followed by calcium acetate in over 45,000 patients (22%). The weighted average dose among all phosphate binders was 6.7 tablets/day. If other combinations of medications have similar average doses, the weighted average total binder dose among all patients would be 7.1 tablets/day (data not shown). 

Prescriptions for 2 or more different phosphate binder medications were documented for over 12,000 patients (5.8%). Table 3 provides the percent of patients documented as receiving the specified medication, and the percentage in parentheses represents the subset of patients who had a prescription for 1 or more additional binders. The most common combination was calcium acetate plus sevelamer (carbonate or hydrochloride), for 2.6% of the patients. Those patients were prescribed a median of 6 calcium acetate tablets plus 6 sevelamer tablets, for a total of 12 phosphate binder tablets per day. 

Since the patient’s phosphate burden could probably increase with each medication added, the potential phosphate binder pills needed to remove that extra phosphate would also increase. Using published values for binding capacities, we calculated the potential increase in the phosphate binder pill burden (Equation 1) due to the most prescribed medications in FKC dialysis clinics (Table 4). Daugirdas et al. [15] calculated that sevelamer, which is considered standard of care, can bind up to 26 mg of phosphate/g; thus, an 800-mg tablet would bind 20.8 mg of phosphate. Calcium carbonate and calcium acetate both bind ~ 45 mg of phosphate/g of compound [15], which is equivalent to 30.0 mg/667 mg tablet of calcium acetate or 22.5 mg per 500 mg tablet of calcium carbonate. A 500-mg Velphoro tablet can bind from 72.5 to 132 mg of phosphate depending on pH [16], and each gram of elemental lanthanum can bind up to 135 g of phosphate [15, 17]. Our analysis suggests that addition of amlodipine, lisinopril, or omeprazole could significantly increase the phosphate binder pill burden, particularly with sevelamer or calcium-based binders. 

## Discussion 

To our knowledge, this is the first database analysis that examines the contribution of phosphate in medications to the overall phosphate burden in HD patients from a large dialysis organization (LDO). It is well known that phosphate control is problematic in dialysis patients, and dietary sources should be carefully monitored. Phosphate intake in HD patients reportedly ranges from ~ 790 to 1,100 mg/day [18, 19, 20]. Considering that (a) ~ 60 – 80% of the dietary phosphate is absorbed in the gut; (b) conventional HD with a high-flux, high-efficiency dialyzer can remove, on average, 30 mmol (900 mg) phosphate during each dialysis performed 3 times weekly; and (c) erythropoietin can reduce phosphate absorption, Takeda suggests that 750 mg of phosphorus intake should be considered as the critical value above which a positive phosphorus balance can occur [1, 18, 21, 22, 23]. That value corresponds to a protein diet of ~ 65 g/day or 0.93 g/kg body weight/day for a 70-kg patient. However, many patients exceed this because of hidden inorganic phosphate (iP) in their diets. 

In this assessment, we identified that individually prescribed medications can contribute substantial amounts of inorganic phosphate to a dialysis patient’s daily phosphate burden, and this varies significantly among manufacturers [13]. Most of the phosphate salt excipients in medications are present in the form of inorganic phosphates – which have higher bioavailability. The formulation (i.e., mono-, di-, or tri-calcium phosphate) determines the degree of solubility and dissociation of the individual salt. Factors such as temperature, pH, and respective concentrations of Ca^2+^ and phosphate of the solutions [24, 25] also play key roles. Calcium phosphate contributed by medications would tend to stay in solid phase at lower temperature, higher pH, and higher concentrations of individual calcium and phosphate proportions. Since most of the medications prescribed in our study contain dibasic calcium phosphate (aka: calcium monohydrogen phosphate, calcium hydrogen phosphate; anhydrous, hemihydrate, or dihydrate), we can speculate that at least 86 – 96% of the calcium salt will be available for absorption [10]. Granted, to obtain actual phosphate absorption, stool phosphate should be measured to determine the fraction that gets absorbed in anuric patients, or urinary phosphate in subjects with normal kidney function. 

Most dialysis patients have several comorbidities and are usually prescribed multiple medications at any given time [26, 27]. Our sample population is no exception, as 66% of patients are diabetic and 27% exhibit cardiovascular issues (Table 1), and the most prescribed drugs were a calcium channel blocker, an ACE inhibitor, and an antihypertensive (Table 2). Based on the median dose, three of the five most prescribed medications in our population could individually contribute up to an additional 112 (amlodipine), 116.2 (lisinopril), and 200 (omeprazole) mg to a patient’s daily phosphate load. Hypothetically, if a patient was prescribed the five most ordered medications seen here at the median daily dose day, the increased daily phosphate load could increase by up to 428 mg, which could require a maximum of 21 additional sevelamer tablets. Consider if four medications containing phosphate – amlodipine, lisinopril, omeprazole, and diclofenac – were combined, then the added phosphate burden would be ~ 766 mg of phosphate /day (112 + 116.2 + 200 + 338), which together with necessary food and drink, would exceed the recommended daily phosphate intake for dialysis patients. 

Another scenario that can be cause for concern is the use of diphenhydramine (Benadryl) to ameliorate itching symptoms commonly experienced by uremic patients. Based on our analysis, diphenhydramine contains a very high phosphate content, contributing 270 mg of phosphate for the median dose prescribed. It is also readily available to patients as an over-the-counter formulation. Thus, for many kidney patients with itching, taking diphenhydramine routinely might significantly contribute to the overall phosphate load. 

Among the prescribed phosphate binders, the weighted average number of phosphate binder tablets/day in this study was 6.7 tablets/day without considering combinations and estimated to be 7.1 tablets/day including combinations. This is slightly lower than the mean (± standard deviation) number of phosphate binder tablets/day of 7.4 ± 4.7 in the U.S. reported by Fissell et al. [30]. Although the mean binder dose observed here falls within the overall range stated in that DOPPS report, there were patients who were prescribed up to 14 binders. This suggests suboptimal serum phosphate control. High serum phosphate levels can lead to, among other issues, increased binder prescription, increased pill burden and low patient adherence. Chiu et al. [28] reported a median daily pill burden in dialysis patients (N = 233) of 19, with some patients being prescribed more than 25 tablets/day (mean: 11 ± 4 medications/day); and phosphate binders contributed to ~ 50% of the daily pill burden. We calculated the worst-case hypothetical increase in phosphate binder burden if the maximum amount excipient phosphate were accounted for in patient’s daily phosphate intake. With three of the top five medications, amlodipine, lisinopril, or omeprazole, the phosphate binder pill burden could increase significantly, particularly with sevelamer or calcium-based binders. Polypharmacy can also adversely impact medication adherence, particularly in dialysis patients. A systematic review reported that, on average, 51% (range: 22 – 74%) of dialysis patients do not adhere to their phosphate binder therapies [29]. In a DOPPS study, high serum levels of phosphate (> 5.5 mg/dL) was associated with non-adherence to phosphate binder prescription, with only 43% of patients in the U.S. reported taking their binders as prescribed [30]. Binder non-adherence also increased with the number of tablets prescribed per day. Nonadherence to prescriptions can, in turn, lead to higher morbidity and mortality [31]. 

There are some limitations to this assessment. First, this is a retrospective review of data, and we cannot determine cause and effect. Second, data on serum phosphate levels, average or median overall pill burden, number of patients on multiple medications, and adherence were not collected. Third, the phosphate content was determined from published information, prescribing information, and manufacturing processes, not laboratory measures; thus, the actual content may vary. Finally, our calculations for estimating the potential increase in phosphate binder use (Table 4) assume that 86 – 100% of the phosphate from medications would be available for absorption, which may not be the case. Thus, our increases in binder numbers may be overestimated. 

Importantly, this is the first database analysis that addresses the potential impact of phosphate content of medications in patients from an LDO. This paper brings to light the importance of “hidden phosphates” in medications and the potential impact on phosphate burden, treatment implications, and medication adherence. Although Cupisti et al. [13] suggests the prevalence of phosphate-containing medications used in CKD patients is low, the problem should not be overlooked. Phosphate binders are prescribed in over 80% of US dialysis patients, and it is all too clear that ~35% of patients are still above KDOQI recommended phosphate target levels [2, 32]. It is also evident that uncontrolled hyperphosphatemia is still a problem [7, 28, 33, 34]. However, rarely, if ever, is phosphate contribution from medications considered as part of the overall phosphate burden. 

The dialysis patient is at an unrecognized disadvantage as he or she is most likely taking numerous medications with different amounts of phosphate from a variety of sources, even for the same pharmaceutical. Phosphate content in medications prescribed to HD patients can substantially contribute to the daily phosphate load and may even exceed the daily recommended dietary phosphate intake in some situations. This unseen phosphate can have a negative impact on the patient’s ability to maintain serum phosphate levels within the target range, as well as on a clinician’s ability to appropriately manage phosphate binder prescriptions. Physicians may also be unaware of concurrent medications being administered or may not realize the phosphate content in the specific formulation form a given manufacturer. Thus, it is imperative that phosphate content in medications be accounted for in the overall daily intake. Healthcare providers should monitor all medications containing phosphate prescribed to dialysis patients, and their sources, in order to minimize risk of uncontrolled hyperphosphatemia and its downstream effects, and poor adherence. 

## Funding 

No funding needed. 

## Conflict of interest 

All authors are employees for Fresenius Medical Care North America. 

**Table 1.** Characteristics of in-center hemodialysis patients with at least 1 prescribed home medication.

Total number of patients (N)	209,811
Age (years) (mean ± SD)	62.5 ± 14.6
Gender (N, %)	
Female	92,822 (44.2)
Male	116,989 (55.8)
Race (N, %)	
Caucasian	124,636 (59.4)
African American	68,549 (32.7)
Asian	5,067 (2.4)
American Indian or Alaska Native	1,797 (0.9)
Native Hawaiian or Pacific Islander	1,498 (0.7)
Hispanic	27,275 (13.8)
Other or unknown	8,264 (3.9)
Comorbidities (N, %)	
Diabetes mellitus (DM)	138,608 (66.1)
Congestive heart failure (CHF)	50,941 (24.3)
Peripheral vascular disease (PVD)	34,549 (16.5)
Cerebrovascular accident (CVA)	10,768 (5.1)
Acute myocardial infarction (AMI)	5,597 (2.7)

**Table 2.** Average phosphate content by frequency of medication prescribed in Fresenius Kidney Care in-center hemodialyisis patients [3, 4, 12, 13, 14].

Ranked by frequency	Medication	Phosphate content/tab (mg)	Median dose (# of tablets per day)	Phosphate contribution by median daily dose (mg)
1	Amlodipine	3.8 – 116.6 dibasic calcium phosphate	1	3.3 – 112
2	Lisinopril	3.6 – 121 dibasic calcium phosphate	1	3.1 – 116.2
3	Clonidine	1.4 – 3.5 dibasic calcium phosphate	2	2.4 – 6.72
4	Acetaminophen	0	4	0
5	Omeprazole	175 – 200 disodium hydrogen phosphate	1	175 – 200
6	Nifedipine	40 dibasic calcium phosphate	1	40
7	Pantoprazole	175 – 200 calcium glycerophosphate	1	175 – 200
8	Tramadol	62 dibasic calcium phosphate	3	160 – 178
9	Esomeprazole	175 – 200	1	175 – 200
10	Sertraline	2.1 – 8.7 dibasic calcium phosphate	1	1.8 – 8.4
11	Glipizide	28 dibasic calcium phosphate	1	24 – 26.9
12	Alprazolam	82.5 dibasic calcium phosphate	2	142 – 158.4
13	Rosuvastatin	1.8 – 3.8 tribasic calcium phosphate	1	1.5 – 3.6
14	Diphenhydramine	150 dibasic calcium phosphate	2	258 – 270
15	Sitagliptin	7.3 – 13.2 dibasic calcium phosphate	1	6.3 – 12.6
16	Paroxetine	0 – 443.7 dibasic calcium phosphate	1	0 – 426
17	Phenytoin	50 dibasic calcium phosphate	2	86 – 96
18	Sildenafil	131 dibasic calcium phosphate	1	112.7 – 125.8
19	Glyburide	0 – 27.6 dibasic calcium phosphate	1	0 – 26.5
20	Estradiol	0	1	0
27	Diclofenac	176 dibasic calcium phosphate	2	302.7 – 338

**Table 3.** Median phosphate binder prescription frequency and dose.

Binder type	Total % of patients (% w/2^nd^ binder)	Median # of tablets/day	Median daily dosage (g)	Standard deviation daily dosage (g)
Calcium acetate	22% (3.7%)	6	4.0	2.3
Calcium carbonate	5.9% (2.0%)	3	3.0	5.3
Lanthanum carbonate	2.6% (0.9%)	3	3.0	1.9
Sevelamer carbonate	26% (4.1%)	6	4.8	3.4
Sevelamer hydrochloride	3.1% (0.5%)	6	4,800	3.2
Sucroferric oxyhydroxide	1.1% (0.07%)	3	1,500	1.2
No binder documented	46%	N/A	N/A	N/A

**Table 4.** Potential maximum increase in daily phosphate binder pill burden (tablets/day rounded to the nearest half pill) with the most prescribed medications in Fresenius Kidney Care clinics.

Medication	Sevelamer (tabs/day)	Calcium carbonate (tabs/day)	Calcium acetate (tabs/day)	Velphoro (tabs/day) ^†^	Lanthanum Carbonate** (tabs/day)
Amlodipine	5.5	5	4	1	1.5
Lisinopril	5.5	5	4	1	1.5
Clonidine	0	0	0	0	0
Acetaminophen	0	0	0	0	0
Omeprazole	9.5	9	6.5	1.5	3

*If the patient is using this product, dietary adjustment may offset need for extra dose; ^†^assumes maximum binding; **assumes 500-mg dose. Calculations assume complete availability of phosphate from medications
